# How feelings of unpleasantness develop during the progression of motion sickness symptoms

**DOI:** 10.1007/s00221-021-06226-1

**Published:** 2021-09-30

**Authors:** A. J. C. Reuten, S. A. E. Nooij, J. E. Bos, J. B. J. Smeets

**Affiliations:** 1 Department of Human Movement Sciences, Vrije Universiteit Amsterdam, Amsterdam, The Netherlands; 2 Human Performance, TNO Soesterberg, Soesterberg, The Netherlands; 3 Department of Human Perception Cognition and Action, Max Planck Institute for Biological Cybernetics, Tübingen, Germany

**Keywords:** Syndrome, Disease progression, Discomfort, Well-being, Self-report, Rating

## Abstract

To mitigate motion sickness in self-driving cars and virtual reality, one should be able to quantify its progression unambiguously. Self-report rating scales either focus on general feelings of unpleasantness or specific symptomatology. Although one generally feels worse as symptoms progress, there is anecdotal evidence suggesting a non-monotonic relationship between unpleasantness and symptomatology. This implies that individuals could (temporarily) feel better as symptoms progress, which could trouble an unambiguous measurement of motion sickness progression. Here we explicitly investigated the temporal development of both unpleasantness and symptomatology using subjective reports, as well as their mutual dependence using psychophysical scaling techniques. We found symptoms to manifest in a fixed order, while unpleasantness increased non-monotonically. Later manifesting symptoms were generally judged as more unpleasant, except for a reduction at the onset of nausea, which corresponded to feeling better. Although we cannot explicate the origin of this reduction, its existence is of importance to the quantification of motion sickness. Specifically, the reduction at nausea onset implies that rating how bad someone feels does not give you an answer to the question of how close someone is to the point of vomiting. We conclude that unpleasantness can unambiguously be inferred from symptomatology, but an ambiguity exists when inferring symptomatology from unpleasantness. These results speak in favor of rating symptomatology when prioritizing an unambiguous quantification of motion sickness progression.

**Supplementary Information:**

The online version contains supplementary material available at 10.1007/s00221-021-06226-1.

Motion sickness is a syndrome of discomfort that may be induced by exposure to a physical or virtual motion stimulus (Cha et al. 2021). Research on the mitigation of motion sickness is gaining interest in particular with respect to autonomous driving (Diels and Bos 2016; Iskander et al. 2019; Jones et al. 2018; Kuiper et al. 2020; Yusof et al. 2020) and virtual reality (Kim et al. 2018; Nooij et al. 2017b; Rebenitsch and Owen 2016; Saredakis et al. 2020). However, to find solutions for mitigating motion sickness, one should be able to quantify it unambiguously.

The Motion Sickness Incidence (MSI), defined as the percentage of people who reach the limit of vomiting during a certain timeframe, has been a popular index to quantify motion sickness in the past (ISO 1997; McCauley et al. 1976; O’Hanlon and McCauley 1973). Although the MSI may be considered the most objective measure, it entirely neglects the wide range of unpleasantness and symptoms encompassing the earlier stages of motion sickness. Therefore, self-report scales that also cover these earlier stages are nowadays an often-used alternative to measure motion sickness. As an alternative to elaborate multi-value questionnaires (Gianaros et al. 2001; Kennedy et al. 1993), single value rating scales (Lawson 2014a) have become particularly popular. For such a report, subjects assign one value on a given scale to indicate their feelings and/or symptoms. After subjects have familiarized themselves with such a scale, they can easily report on it within a second, with minimal interference on any task performed, and allowing repeated application within experimental sessions, even with eyes closed. This paper limits its scope to this specific type of numerical scales. These scales can largely be grouped into two categories: scales questioning how bad someone feels, here termed unpleasantness, or scales based on the symptomatology one experiences. In this paper, we address the relationship between these two types of scales.

Scales rating unpleasantness use a severity grading to report on a general feeling of malaise (Draper et al. 2001; Jones et al. 2018; Keshavarz and Hecht 2011; Lawther and Griffin 1986; Reason and Graybiel 1970; Turner and Griffin 1999). They often use magnitude estimation, anchored with endpoints ranging from feeling fine to feeling absolutely dreadful. One example, that will be analyzed in the current context, is the Fast Motion sickness Scale (FMS), in which observers give verbal ratings of experienced motion sickness on a 20-point scale ranging from 0 (no sickness) to 20 (frank sickness) (Keshavarz & Hecht 2011). On the other hand, scales rating symptomatology often include a numerical characterization which is based on the observation that different classes of symptoms generally progress in a fixed order over time. Although bodily symptoms like flushing, stomach awareness, and dizziness often vary between people, this class of symptoms is typically followed by nausea, retching and ultimately vomiting (Bos et al. 2005; Lawson 2014b; Reason and Brand 1975; Reason and Graybiel 1970). This allows these classes to be given incremental values, possibly with a grading for the experienced severity within a symptom class (Bos et al. 2005; Donohew and Griffin 2004; Golding et al. 2001, 2003; Golding and Kerguelen 1992; Hemingway 1975; McCauley et al. 1976). The largest refinement is provided by the MISC (Bos et al. 2005) as given in Table 1. Different from its original naming will we refer to this scale as the Motion Illness Symptoms Classification.

**Table 1:** The Motion Illness Symptoms Classification (MISC) used to assess motion sickness symptomatology (Bos et al. 2005)

Class description	MISC
No problems	0
Some discomfort, but no specific symptoms	1
Dizziness, cold/warm, yawning, headache, tiredness, sweating, stomach/throat awareness, burping, blurred vision, salivation, … but no nausea	
Vague	2
Little	3
Rather	4
Severe	5
Nausea	
Little	6
Rather	7
Severe	8
Retching	9
Vomiting	10

With both types of scales often being used in research on motion sickness, there is a surprising lack of knowledge on how feelings of unpleasantness develop during the progression of motion sickness symptoms. Intuitively, one feels worse as symptoms progress, which is supported by the high positive correlations observed between measures of unpleasantness and symptomatology (Bos et al. 2005; D’Amour et al. 2017; Keshavarz and Hecht 2011; Nooij et al. 2017a, b; Reason and Graybiel 1970). Yet, such correlations hide possible local deviations of a monotonic relationship. If unpleasantness ratings were found to decrease with ongoing motion stimulation, this would trouble an unambiguous measurement of motion sickness progression. Anecdotal evidence indeed suggests unpleasantness to increase non-monotonically with symptom progression. To illustrate, vomiting is generally considered the final manifesting symptom, yet also reported to offer relief of misery (Dobie 2019; Lackner 2014; Leung and Hon 2019). Additionally, one study reported specific decreases in unpleasantness ratings during ongoing motion stimulation, also suggesting the presence of a non-monotonic relationship (Reason and Graybiel 1970). These two examples provide reason to assume that rating how bad someone feels may not be equivalent to rating how close someone is to the point of vomiting.

In the present study, we therefore systematically explored the relationship between unpleasantness and symptomatology during the progression of motion sickness. Firstly, we focus on how unpleasantness and symptomatology develop for up to 30 min of motion stimulation. We there explicitly investigate if they increase monotonically with the progression of motion sickness over time. Secondly, we focus on the relationship between unpleasantness and symptomatology, answering the question: do we consistently feel worse as symptoms progress?

## Methods

### Temporal development of unpleasantness and symptomatology

#### Data collection

In this first part, we investigate how unpleasantness and symptomatology develop with the progression of motion sickness. To do so, we (re-)analyzed motion sickness ratings collected during five previously published experiments (Exp 1 = Nooij et al. 2017b; Exp 2 = Nooij et al. 2017a; Exp 3 = Nooij et al. 2021; Exp 4 = Bos et al. 2005; Exp 5 = Bos 2015) and two additional experiments to be published later (Exp 6–7). In all experiments, subjects were exposed to either physical or virtual motion for a maximum duration of 30 min and indicated their level of unpleasantness or symptomatology at regular intervals (two to five minutes). Unpleasantness was assessed in Exp 1–3 using the FMS, whilst symptomatology was assessed in Exp 4–7 using the MISC. The provocative stimulation was aborted when a subject reported a FMS class of ≥ 15 or a MISC class of ≥ 7, except for Exp 4 that used no stop-criterion. All experiments (except for Exp 3) consisted of multiple provocative sessions, which were presented on separate days. Additional experimental details are summarized in Supplementary Table S1.

#### Data analysis

We analyzed the FMS ratings from 58 subjects performing a total of 132 sessions with at least two ratings within each session, and MISC ratings from 148 subjects performing a total of 528 sessions with at least two ratings within each session. For all scale ratings, we analyzed the difference in rated class between two consecutive ratings, which we will further refer to as a rating transition. We first determined the number of observed transitions between two classes, and subsequently calculated the proportion of cases in which the rating after a certain class remained constant, increased, or decreased. Our null hypothesis is a monotonic increase of unpleasantness and symptomatology with the progression of motion sickness over time, implying that their respective ratings should increase or remain constant. Decreases in ratings might occur due to random fluctuations in rating, and thus should be infrequent and evenly distributed over the whole range of the scale.

To promote a comparison with the normalized results for unpleasantness on the psychophysical scaling tasks (see next section), we rescaled the FMS to describe the temporal development of unpleasantness to range from 0 “no sickness” to 1 “frank sickness”, which we refer to as FMS’.

### Relationship between unpleasantness and symptomatology

#### Data collection

In the second part, we assessed the relationship between unpleasantness and symptomatology. This part was performed in Exp 6 and 7, in which subjects performed a psychophysical scaling task before and/or after the last provocative session of the experiment.

In Exp 6, subjects judged the level of unpleasantness associated with each MISC class using *magnitude estimations* (MAG) as originally used for the ratio scaling of psychophysical stimuli, such as the brightness of light (Stevens 1956) or social phenomena (Kuennapas and Wikstroem 1963; Lodge 1981; Venrooij et al. 2015). We here asked subjects to draw lines whose lengths represented the level of unpleasantness they associated with each MISC class description (1 to 10). We only provided the descriptions, without referring to the numerical values corresponding to the classes. We provided two A4 papers in landscape orientation, with a horizontal 10.5 cm reference line at the top of each page. This line represented the unpleasantness for MISC 6, whose description was printed below the line. In addition, four or five other descriptions were printed below, which we asked subjects to judge by drawing a line. We explained subjects that drawing a line twice the length of the reference line, would imply twice the amount of unpleasantness as compared to the reference symptom (i.e., feeling a little nauseated). Lines could be of any length, if needed consisting of multiple line segments. The class descriptions were randomized in four different orders. We let subjects perform this task both before the first session and after the last, to investigate whether exposure to a provocative motion affected the judgements.

In Exp 7, we investigated whether the choice of reference class affected the judgements. We therefore repeated the MAG task of Exp 6 using class description MISC 4 instead of MISC 6 as the reference. In addition, we investigated whether the type of psychophysical task affected the judgements by letting subjects perform a *two-alternative forced choice* (2AFC) task (Thurstone 1927). In this 2AFC task, we presented subjects two MISC class descriptions and asked them “which of these two symptoms do you consider most unpleasant?”. Ignoring the order of the two descriptions within each comparison, this resulted in 45 comparisons that were presented in a random order using a computer. Both the MAG and the 2AFC task were performed once, either before the first session, or after the last. The order of tasks was counterbalanced between subjects.

In Exp 6–7, we asked subjects to rate their experienced unpleasantness directly after a session on a *visual analogue scale* (VAS). Whilst the MAG and 2AFC tasks asked subjects to imagine how they would feel when experiencing the symptoms described, and were thus made independent of a motion stimulus, the VAS rating allowed for a direct comparison of the experienced unpleasantness and the highest MISC rating given during that session. The VAS consisted of a 12 cm line segment with endpoints “very unpleasant” and “very pleasant”. Subjects marked their judgement on this line and also indicated the main reason of their experienced unpleasantness, by choosing one of the following categories: motion sickness, physical stress, temperature, smell, sound, boredom, other, and not applicable.

#### Data analysis

To equalize the scale range between subjects and allow for an optimally balanced comparison of the three tasks, we normalized all psychophysical ratings. For the MAG task, we first measured the drawn line length (*L*) for each question with a ruler. We then determined the normalized MAG ratings for each subject using their shortest and longest drawn line, giving ${\text{MAG}}\; = \;\left( {L\; - \;L_{\min } } \right)/\left( {L_{\max } \; - \;L_{\min } } \right)$. We add subscripts 6 and 4 to refer to the reference used: MAG_6_ for the task using MISC 6 (*n* = 30) and MAG_4_ for the task using MISC 4 (*n* = 79). For the 2AFC task (*n* = 83), we first counted the number of times (*C*) a subject chose a MISC class as the most unpleasant. We then determined the normalized 2AFC ratings for each subject using the counts of the classes they had rated least and most unpleasant, giving ${\text{2AFC}}\; = \;\left( {C\; - \;C_{\min } } \right)/\left( {C_{\max } \; - \;C_{\min } } \right)$. For the VAS task (*n* = 107), we first measured the distance up to the mark that each subject had drawn *(V)*. We then determined the normalized VAS rating for each subject by dividing this distance by the total line length, giving VAS = *V*/12.

Five subjects in Exp 6 and six subjects in Exp 7 did not perform all rating tasks. There were two subjects who misinterpreted the MAG_4_ task and reversed the sign for their line drawings (i.e., MISC 1 or 2 receiving 1 and MISC 9 or 10 receiving 0). They performed as expected in their 2AFC ratings. For these subjects, we replaced the MAG_4_ ratings by 1-MAG_4_. Due to an administrative error, two subjects performed the 2AFC task twice. We averaged their responses in the data analysis.

Our null hypothesis is a monotonic increase in unpleasantness with increasing symptom progression. To test for possible reductions in unpleasantness with increasing symptom progression, we compared the MAG and 2AFC ratings for all pairs of successive MISC classes using one-sided Wilcoxon Signed Rank tests with Bonferroni correction (*α* = 0.0056). For the VAS ratings, we followed the same procedure but with one-sided Mann–Whitney *U *tests instead (*α* = 0.0063).

Regarding the visual presentation of data, error bars are generally plotted in the direction of the axes. Because some data allowed for a within-subject comparison of ratings (Figs. 2a and 4a), we used the opportunity to determine the interquartile ranges in directions that take the within-subject characteristics into account: along the identity line and perpendicular to that. The rotation applied to these data resulted in the displacement of some medians due to an asymmetric distribution of data points (see Supplementary Fig. S1).

## Results

### Temporal development of unpleasantness and symptomatology

**Fig. 1:**
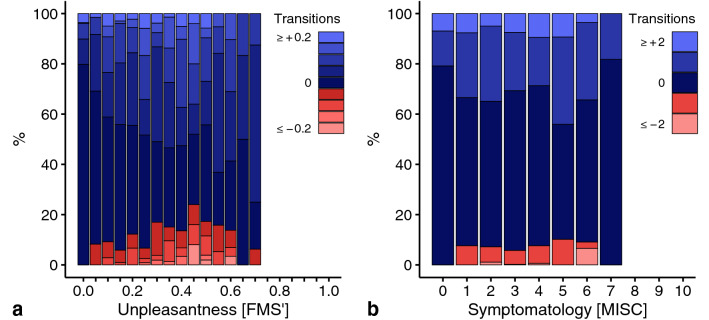
Overview of the transitions in consecutive ratings during ongoing motion stimulation. Colors indicate whether the transitions are consistent with a monotonic increase (bluish) or not (reddish). Sessions were generally terminated once subjects reached FMS’ 0.75 or MISC 7. **a** Unpleasantness ratings using the FMS’. **b** Symptomatology ratings using the MISC. Contrary to the FMS’ is there no clear peak indicating non-monotonic behavior

To investigate the temporal development of unpleasantness and symptomatology, we analyzed consecutive ratings collected on, respectively, the FMS’ and MISC scale during ongoing motion stimulation in Exp 1–7. Note that the number and distribution of decreasing rating transitions tells whether unpleasantness and symptomatology increase monotoncially with the progression of motion sickness over time. Figure 1 shows the distribution of transitions for the FMS’ and MISC. Whereas decreases accounted for only 4% of all transitions for the MISC, this proportion was doubled (8%) for the FMS’. In addition, where the decreases were distributed evenly over all classes of the MISC (for MISC 1 to 6 between 6 and 10%), the FMS’ decreases peaked (24%) in the central area of the unpleasantness scale. Moreover, in 45% of all sessions rated using the FMS’, one or multiple decreases occurred, which only applied to 25% of all sessions rated using the MISC. The number of transitions in consecutive ratings for both types of scales is presented in Supplementary Fig. S2.

These results show that decreases in unpleasantness ratings occur more frequently, and are moreover linked to the center of the scale, compared to the decreases in symptomatology ratings. This suggests that subjects temporarily feel better during motion sickness progression, which is an indication of a non-monotonic dependence of unpleasantness on symptom progression.

### Relationship between unpleasantness and symptomatology

**Fig. 2:**
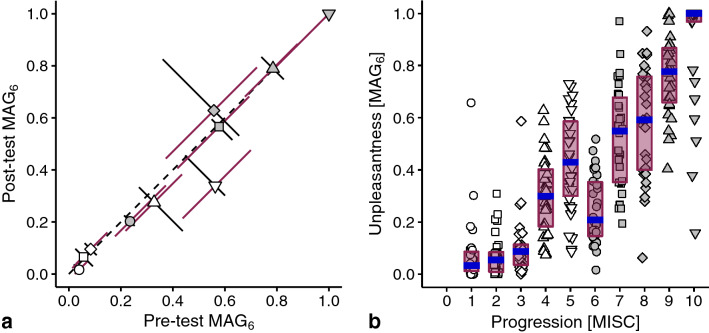
The unpleasantness of the various MISC classes rated using magnitude estimation with MISC 6 as a reference (MAG_6_). **a** Comparison of median ratings given before and after the last exposure to a provocative motion. The symbols correspond to MISC classes (see panel **b**). The error bars indicate interquartile ranges. They express the between-subject variability in pre-test/post-test difference (black error bars) and in overall ratings (magenta error bars). **b** Individual MAG_6_ ratings (symbols), medians (in blue), and interquartile ranges (magenta bars) of the corresponding unpleasantness of 10 MISC class descriptions. Horizontal jitter is added to the individual ratings for distinguishability

We collected information on how unpleasantness corresponds with each of the MISC classes using three psychophysical scaling tasks. In Exp 6, subjects performed the MAG task using MISC 6 as a reference (MAG_6_) both before and after a provocative session. The results show that the experience of motion sickness did not affect the judgements (Fig. 2a). The ratings for most classes are well reproducible, with MISC 4, 5, and 8 showing the largest variability between measurements. Given that all perpendicular error bars overlap the identity line, we pooled the pre-test and post-test ratings in further analyses. Our main observation is that unpleasantness generally increased with symptom progression, with a noticeable exception for the rating on MISC 6, at the onset of nausea (Fig. 2b). The only comparison where the unpleasantness was lower on a successive MISC class, was for MISC 6 compared to MISC 5 (*α* = 0.0056, *p* < 0.001; *r* =  − 0.61).

**Fig. 3:**
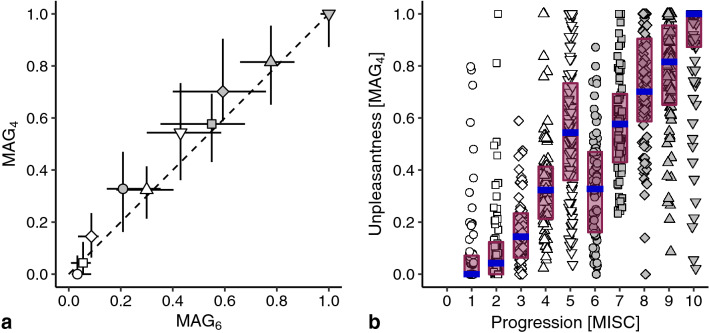
The unpleasantness of the various MISC classes rated using magnitude estimation with MISC 4 as a reference (MAG_4_). **a** Comparison of median ratings across subjects with those rating MAG_6_ in the first experiment. Horizontal error bars represent interquartile ranges for MAG_6_ and vertical error bars interquartile ranges for MAG_4_. **b** Individual MAG_4_ ratings, medians, and interquartile ranges. Further details as in Fig. 2

To investigate whether the reduction at MISC 6 was not just a reflection of the choice of reference, we let subjects perform the MAG task with MISC 4 (MAG_4_) as the reference in Exp 7. The results show that the ratings do not depend strongly on the reference used (Fig. 3a). Although MAG_4_ ratings were slightly larger than MAG_6_ ratings, the error bars for all MISC classes overlap the identity line. Most importantly, Fig. 3b shows the same exception of the increase in unpleasantness at MISC 6. The tests indeed showed that the unpleasantness at MISC 6 was significantly reduced compared to that on MISC 5 (*α* = 0.0056, *p* < 0.001; *r* =  − 0.38).

**Fig. 4:**
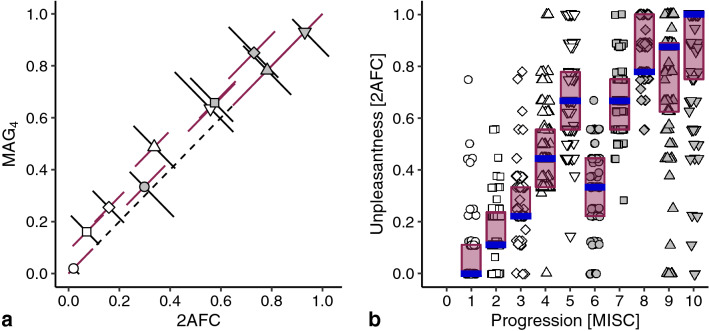
The unpleasantness of the various MISC classes rated using a 2-alternative forced choice task (2AFC). **a** Comparison of median within-subject MAG_4_ and 2AFC ratings. **b** Individual 2AFC ratings, medians, and interquartile ranges. Further details as in Fig. 2

We then wanted to confirm that the obtained results were not restricted to the used rating technique, for which subjects performed the 2AFC task in Exp 7 as well. The normalized 2AFC ratings were slightly larger in unpleasantness than the MAG_4_ ratings (Fig. 4a), but as all perpendicular error bars overlap the identity line, we consider the ratings of these two tasks equivalents. This is substantiated in Fig. 4b, which again demonstrates an exception of the increase in unpleasantness at MISC 6. This reduction in unpleasantness at the transition from MISC 5 to MISC 6 tested significant (*α* = 0.0056, *p* < 0.001; *r* =  − 0.56). In contrast to the data in Figs. 2b and 3b, the statistical analysis of the data in Fig. 4b showed a second decrease: although the median of MISC 9 is higher than MISC 8, there was a significant reduction in unpleasantness from MISC 8 to MISC 9 (*α* = 0.0056, *p* = 0.0053; *r* =  − 0.22).

**Fig. 5:**
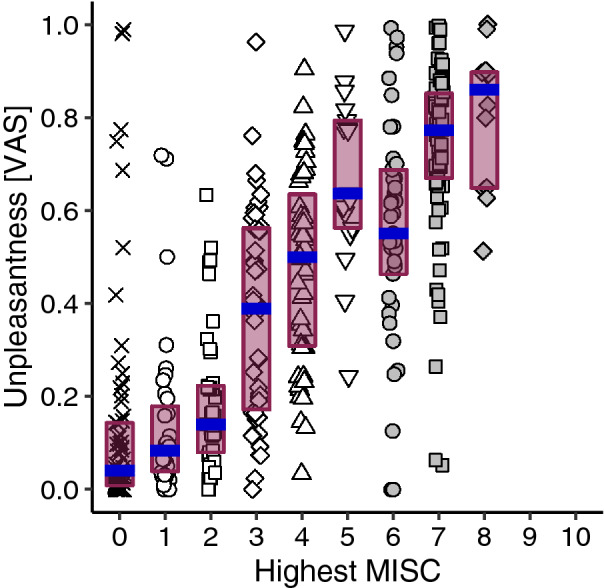
The relationship between the reported unpleasantness experienced during a provocative session and the highest rated MISC class during that session. Further details as in Fig. 2b

In contrast with the MAG and 2AFC tasks, our last comparison with the VAS ratings in Exp 6–7 on the experienced unpleasantness during a provocative session allowed for a direct comparison with the symptomatology rated during that session. When all normalized VAS ratings obtained after sessions are plotted against their highest reported MISC ratings within sessions (Fig. 5), we observe a pattern of results that is very similar to those in Figs. 2b, 3b, and 4b. However, this apparent reduction of unpleasantness at MISC 6 was not significant (*α* = 0.0063,* p* = 0.0514). We established that motion sickness was generally the main cause of unpleasantness during the sessions (see Supplementary Fig. S3).

## Discussion

To facilitate research on mitigating motion sickness, we here compared two major categories of rating scales: those measuring either general unpleasantness or specific symptomatology. We found that during ongoing stimulation, symptoms manifested in a fixed order, while unpleasantness appeared to increase non-monotonically (Fig. 1). Using psychophysical scaling techniques, we then showed that although symptoms manifesting later were generally judged as more unpleasant, there was an exception at the onset of nausea. At this point, subjects systematically indicated that little nausea corresponded to feeling better compared to any severe pre-nausea symptom. We found that this reduction in unpleasantness was independent of a recent episode of motion sickness (Fig. 2.), the choice of reference in magnitude estimations (Fig. 3), the type of rating task (Fig. 4), and was present on visual inspection when considering the experienced unpleasantness within a provocative session (Fig. 5).

A limitation of our data is that the unpleasantness ratings shown in Figs. 2, 3, and 4 were not obtained during exposure to a provocative motion, and thus reflect estimates of unpleasantness based on personal histories. Given that the formulation of the symptoms in the MISC scale at MISC 5 (various severe symptoms) might sound less pleasant than the ‘little nausea’ of MISC 6, such predictions might be biased. Two aspects of our data invalidate this reasoning. Firstly, the ratings obtained *after* a provocative motion did not differ from those obtained *before*: MISC 6 was judged less unpleasant than MISC 5 (gray disc to the left and below the downward pointing triangle in Fig. 2a). Secondly, we observed a similar reduction of unpleasantness at MISC 6 in an experiment where we directly compared motion induced unpleasantness and symptomatology (Fig. 5). The reduction in this comparison is slightly smaller than that in Figs. 2, 3, and 4, which is presumably due to the fact that subjects judged the unpleasantness of the whole session in Fig. 5, rather than that of the highest MISC value they rated (which we used as the independent variable). Therefore, those reaching MISC 6 likely having suffered from the symptoms associated with MISC 5 too.

The anomaly in the otherwise monotonic relationship between unpleasantness and symptom progression concerns MISC classes 5 and 6. Looking at Fig. 1b, these classes also show the largest relative number of decreases, which might raise the question whether the order of MISC classes is appropriate. We believe it is, as over 80% of the rating transitions for these classes were still those of no change or an increment of 1 class. Furthermore, the number of decreases is in the same order of magnitude as those of other MISC classes, suggesting that these decreases are due to inaccuracies in the reports. Hence, it makes most sense to conclude that we do not consistently feel worse as symptoms progress, which answers the main question we explored in this paper. Our study located this specific decrease in unpleasantness at the onset of nausea. Yet, we would like to stress that we replicated the general increase of unpleasantness with symptom progression (Bos et al. 2005; D’Amour et al. 2017; Keshavarz and Hecht 2011; Nooij et al. 2017b; Reason and Graybiel 1970). Our findings fit well in the context of an earlier study reporting a general increase in unpleasantness during ongoing stimulation, but with temporary decreases in those ratings (Reason and Graybiel 1970). Those decreases mainly occurred in the central range of the unpleasantness scale, in alignment with our own observations in which unpleasantness decreased midway the progression of motion sickness symptoms. Also our observation that several subjects judged the unpleasantness of MISC 10 as less than other classes, is in line with the reports of decreasing unpleasantness after vomiting (Dobie 2019; Lackner 2014; Leung and Hon 2019). Further validation of this latter issue is impeded by the fact that our experimental sessions generally stopped at MISC 7 (i.e., before vomiting).

Despite our observation that unpleasantness and symptomatology ratings go hand in hand, the anomaly at the onset of nausea shows that they are two different constructs in the quantification of motion sickness. The question now remains how to explain the observed unpleasantness reduction at nausea onset. We believe that the simplest explanation concerns a cessation of previous symptoms with the introduction of a new class of symptoms. From personal histories, it then makes sense that feeling a little nauseated is less bad than suffering from severe headaches or dizziness, as these latter symptoms more severely impact daily functioning. However, we cannot substantiate this idea because the MISC is not informative on the cessation of individual classes of symptoms during motion sickness progression nor has such information been reported in the literature.

Our results indicate that there is a risk associated with a rating of unpleasantness when wanting to prevent from vomiting during a provocative exposure. Subjects will report to suddenly feel better when progressing from MISC 5 to 6, suggesting that their distance to the point of vomiting increases, whereas they are actually getting closer to that point. We therefore consider a rating of symptomatology more relevant when it is important to prevent individuals from reaching the point of vomiting. For example, in fully automated car driving, automated processes could for instance adjust the driving style of the self-driving car from sporty to relaxed when an occupant indicates to feel slightly nauseated. On the other hand, a rating of unpleasantness is still more useful when testing the attractiveness of a commercial device, for example, of a game played in virtual reality. In any case, we want to caution for a comparison of studies that have employed the two different types of rating scales, as we believe that they cannot one-to-one be compared in terms of motion sickness progression level. After all, we here demonstrated that rating how bad someone feels is not the equivalent of rating how close someone is to the point of vomiting.

To conclude, the non-monotonic dependence of unpleasantness on symptom progression implies that each class of symptoms can be associated with a single unpleasantness rating, while unpleasantness ratings in the center of the scale are associated with multiple classes of symptoms. This effectively means one can predict unpleasantness from symptomatology, while one cannot unambiguously determine symptomatology from measurements of unpleasantness. In Table 2, we present the predicted feelings of unpleasantness corresponding with each class of MISC symptoms, which we have determined by averaging the obtained within-subject MAG and 2AFC data. To come to our overall conclusion, we believe that our results favor a rating of symptomatology when prioritizing an unambiguous quantification of motion sickness progression.

**Table 2:** Conversion table of the predicted median unpleasantness scores from MISC classes denoting symptom progression (*n* = 109)

MISC	Unpleasantness (median)	95% CI
1	0.02	0.00, 0.04
2	0.11	0.08, 0.11
3	0.19	0.16, 0.21
4	0.39	0.36, 0.42
5	0.58	0.54, 0.61
6	0.31	0.29, 0.33
7	0.60	0.58, 0.63
8	0.76	0.72, 0.80
9	0.77	0.75, 0.82
10	0.94	0.89, 0.98

95% confidence intervals (CI) are calculated using bias-corrected and accelerated bootstrapping (*n* = 2500)

## Supplementary Information

Below is the link to the electronic supplementary material.

Supplementary file1 (DOCX 1730 KB)

Supplementary file2 (PDF 11 KB)

Supplementary file3 (PDF 15 KB)

## Data Availability

All code and data are publicly available via the Open Science Framework and can be accessed at https://osf.io/ybw7d/. These materials are licensed under the Creative Commons Attribution-ShareAlike 4.0 International License.
