# Supplementary material for: How feelings of unpleasantness develop during the progression of motion sickness symptoms
Source: Exp Brain Res. 2021 Sep 30;239(12):3615–24. doi: 10.1007/s00221-021-06226-1 (PMC8599357; doi:10.1007/s00221-021-06226-1)
Supplement: Supplementary file 1 — Supplementary file1 (DOCX 1730 KB) [file 221_2021_6226_MOESM1_ESM.docx]

How feelings of unpleasantness develop during the progression of motion sickness symptoms

A. J. C. Reuten^1, 2^*, S. A. E. Nooij^2, 3^, J. E. Bos^2, 1^, J. B. J. Smeets^1^

^1^ Department of Human Movement Sciences, Vrije Universiteit Amsterdam, The Netherlands

^2^ Human Performance, TNO Soesterberg, The Netherlands

^3^ Department of Human Perception Cognition and Action, Max Planck Institute for Biological Cybernetics, Tübingen, Germany

*[a.j.c.reuten@vu.nl](mailto:a.j.c.reuten@vu.nl)

ORCIDs

Reuten: 0000-0003-4641-4180

Bos: 0000-0002-1494-6804

Smeets: 0000-0002-3794-0579

Supplementary Information

Experimental details

|  | | | | | |
| --- | --- | --- | --- | --- | --- |
| **Table S1** Details of the seven experiments which data was (re-)analyzed in this paper | | | | | |
| **Exp** | **Reference** | **Motion stimulus** | **n** | **# sessions** | **Duration (minutes)** |
| 1 | Nooij, Pretto, Oberfeld, et al., 2017 | Visual  yaw rotation | 18 | 4 | 20 |
| 2 | Nooij, Pretto, & Bülthoff, 2017 | Visual  yaw rotation | 21 | 2 | 20 |
| 3 | Nooij et al., 2021 | Visual  yaw rotation | 19 | 1 | 20 |
| 4 | Bos et al., 2005 | Physical  simulated ship motion | 24 | 3 | 30 |
| 5 | Bos, 2015 | Physical  off-vertical axis rotation | 18 | 4 | 20 |
| 6 | - | Physical  vertical oscillations | 30 | 3 | 20 |
| 7 | - | Physical  horizontal oscillations | 84 | 3 | 20 |

The effect of coordinate system on location of the median

|  |  |
| --- | --- |
| 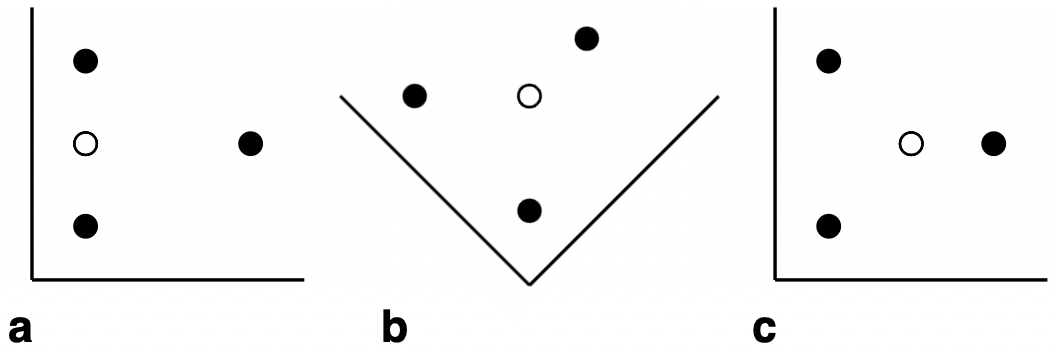 | |
| **Fig. S1** Example of the displacement of medians after rotation of data points. **a.** Three data points (filled) with their median (open). **b.** The same three data points after 45º degree rotation with their median. **c.** Panel b after -45º rotation: the data points are at the same position as in **a**, but the median is at a different position | |

Overview of all observed transitions between consecutive ratings

|  |
| --- |
|  |
| **Fig. S2** Data underlying Fig. 1 of the main text. Overview of transitions between ratings taken at consecutive (k versus k+1) timepoints during ongoing stimulation. **a.** FMS’ ratings on unpleasantness. **b.** MISC ratings on symptomatology. Diagonal cells contain the number of unchanging ratings. The shading of other cells represents the fraction of the off-diagonal transitions in that column |

Reported causes of unpleasantness

|  |  |
| --- | --- |
| **** | |
| **Fig. S3** Main factor contributing to the experienced unpleasantness in response to a provocative motion | |

References

Bos, J. E. (2015). Less sickness with more motion and/or mental distraction. *Journal of Vestibular Research*, *25*(1), 23–33. https://doi.org/10.3233/VES-150541

Bos, J. E., MacKinnon, S. N., & Patterson, A. (2005). Motion Sickness Symptoms in a Ship Motion Simulator: Effects of Inside, Outside, and No View. *Aviation, Space, and Environmental Medicine*, *76*(12), 1111–1118.

Nooij, S. A. E., Bockisch, C. J., Bülthoff, H. H., & Straumann, D. (2021). Beyond sensory conflict: The role of beliefs and perception in motion sickness. *PLOS ONE*, *16*(1), e0245295. https://doi.org/10.1371/journal.pone.0245295

Nooij, S. A. E., Pretto, P., & Bülthoff, H. H. (2017). *Why is vection making you sick? Cognitive factors in Visually Induced Motion Sickness* [Presentation]. 6th International conference on Visually Induced Motion Sensations (VIMS 2017), Toronto, Canada.

Nooij, S. A. E., Pretto, P., Oberfeld, D., Hecht, H., & Bülthoff, H. H. (2017). Vection is the main contributor to motion sickness induced by visual yaw rotation: Implications for conflict and eye movement theories. *PLOS ONE*, *12*(4), e0175305. https://doi.org/10.1371/journal.pone.0175305
