# Supplementary figures and images for: How feelings of unpleasantness develop during the progression of motion sickness symptoms
Source: Exp Brain Res. 2021 Sep 30;239(12):3615–24. doi: 10.1007/s00221-021-06226-1 (PMC8599357; doi:10.1007/s00221-021-06226-1)

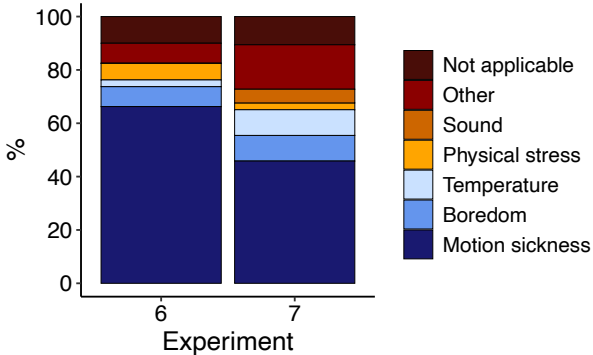

Supplement: Supplementary file 3 — Supplementary file3 (PDF 15 KB) [file 221_2021_6226_MOESM3_ESM.pdf]
