# Supplementary material for: How feelings of unpleasantness develop during the progression of motion sickness symptoms
Source: Exp Brain Res. 2021 Sep 30;239(12):3615–24. doi: 10.1007/s00221-021-06226-1 (PMC8599357; doi:10.1007/s00221-021-06226-1)
Supplement: Supplementary file 2 — Supplementary file2 (PDF 11 KB) [file 221_2021_6226_MOESM2_ESM.pdf]

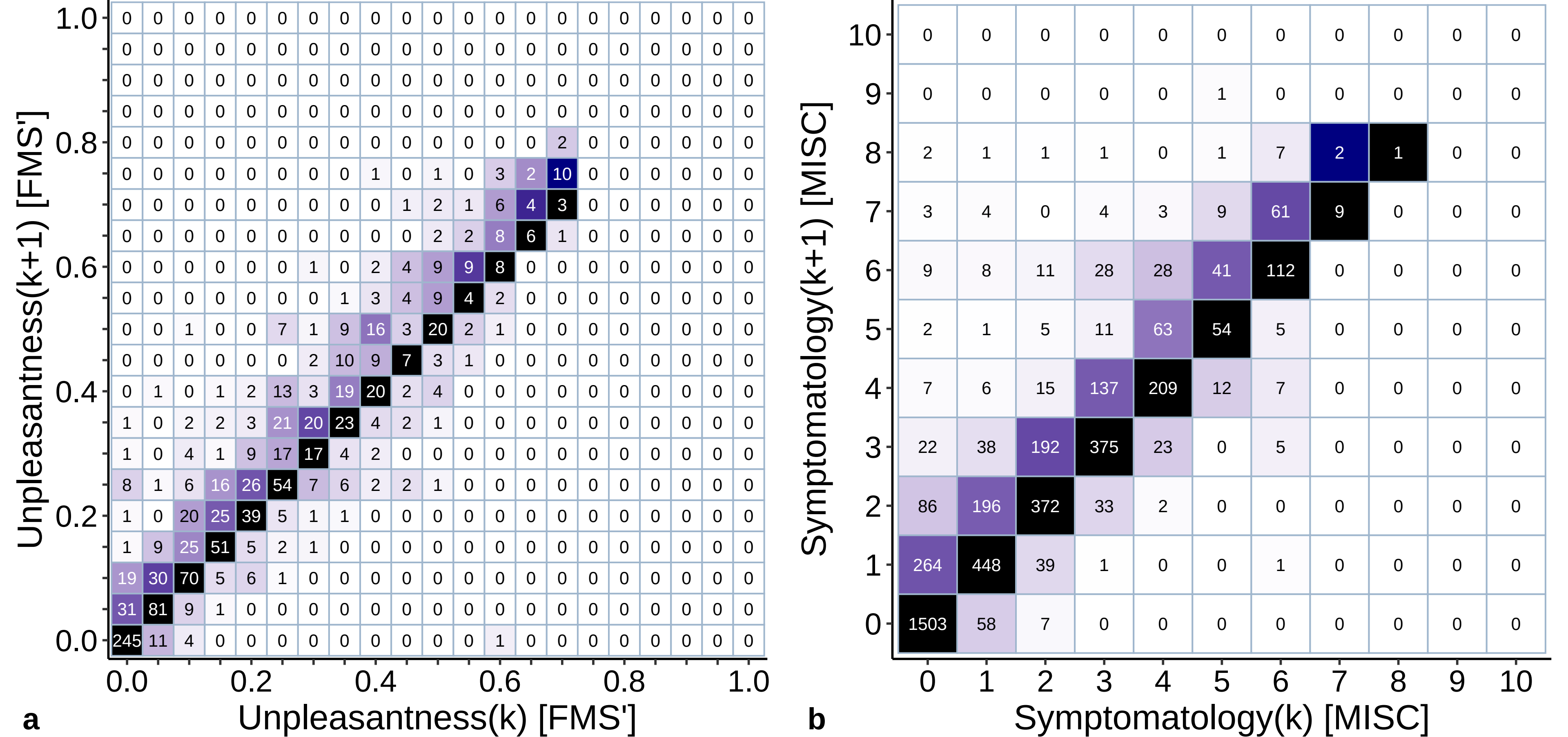
a
Unpleasantness(k+1) [FMS']
Unpleasantness(k) [FMS']
0.0
0.2
0.4
0.6
0.8
1.0
b
Symptomatology(k+1) [MISC]
Symptomatology(k) [MISC]
0
1
2
3
4
5
6
7
8
9
10
